# Theory of sigma bond resonance in flat boron materials

**DOI:** 10.1038/s41467-023-37442-8

**Published:** 2023-03-31

**Authors:** Lu Qiu, Xiuyun Zhang, Xiao Kong, Izaac Mitchell, Tianying Yan, Sung Youb Kim, Boris I. Yakobson, Feng Ding

**Affiliations:** 1 Center for Multidimensional Carbon Materials, Institute for Basic Science (IBS), Ulsan, 44919 Republic of Korea; 2 Department of Materials Science and Engineering, Ulsan National Institute of Science and Technology, Ulsan, 44919 South Korea; 3 College of Physical Science and Technology, Yangzhou University, Yangzhou, 225009 PR China; 4 State Key Laboratory of Information Functional Materials, 2020 X-Lab, ShangHai Institute of Microsystem and Information Technology, Chinese Academy of Sciences, Shanghai, 200050 PR China; 5 Institute of New Energy Material Chemistry, School of Materials Science and Engineering, Nankai Univeristy, Tianjin, 300350 PR China; 6 Department of Mechanical Engineering, Ulsan National Institute of Science and Technology, Ulsan, 44919 South Korea; 7 Department of Materials Science and NanoEngineering, Rice University, Houston, TX 77005 USA; 8 Faculty of Materials Science and Engineering & Institute of Technology for Carbon Neutrality, Shenzhen Institute of Advanced Technology, Chinese Academy of Sciences, 1068 Xueyuan Avenue, Shenzhen, 518055 PR China

**Keywords:** Quantum chemistry, Two-dimensional materials

## Abstract

In chemistry, theory of aromaticity or π bond resonance plays a central role in intuitively understanding the stability and properties of organic molecules. Here we present an analogue theory for σ bond resonance in flat boron materials, which allows us to determine the distribution of two-center two-electron and three-center two-electron bonds without quantum calculations. Based on this theory, three rules are proposed to draw the Kekulé-like bonding configurations for flat boron materials and to explore their properties intuitively. As an application of the theory, a simple explanation of why neutral borophene with ~1/9 hole has the highest stability and the effect of charge doping on borophene’s optimal hole concentration is provided with the assumption of σ and π orbital occupation balance. Like the aromaticity theory for carbon materials, this theory greatly deepens our understanding on boron materials and paves the way for the rational design of various boron-based materials.

## Abstract (web summary)

Here, the authors present a resonance theory to describe the bonding configuration of flat boron materials without quantum calculation. Like aromaticity theory in carbon, it allows to intuitively understand the stability and properties of boron-related materials

## Introduction

Recently, new boron materials including planar boron clusters^1–4^, boron cages^5,6^, boron nanotubes^7,8^, and monolayer boron sheets (borophene)^9–12^, have attracted significant attention. A key feature of these boron isomers is the preference for hexagonal holes within their triangular lattices^6,11,13,14^. Based on extensive quantum calculations, Tang and coauthors proposed that the high stability of boron triangular sheets with holes, compared to the triangular boron lattice, can be attributed to a π-to-σ self-doping mechanism^15^. Although extensive computational studies have been dedicated to exploring the electronic structures of these boron materials^15–18^, theoretical understanding of the bonding configurations of these materials is still lacking.

In the development of organic chemistry, the theory of aromaticity, which allows us to understand the bonding of the organic materials intuitively without performing extensive quantum calculations, plays a central role in materials design and synthesis. Even though today’s quantum calculations can give more accurate results, the lack of a clear chemical picture or intuitive understanding of the computational results made them hard to be used in materials design and synthesis. Therefore, theories of bonding that can predict the properties and stabilities of a large class of materials without quantum calculations, are always highly desirable for materials development.

Historically a three-center two-electron (3c-2e) bond model^19^ derived from the octet rule has been adopted to explain the unusual geometry and the high stability of electron-deficient boron materials^20–22^. The 3c-2e bond in boron system was considered as a result of bond resonance, and the concept of aromaticity has been borrowed from carbon materials to explain the exceptional stability of boron polyhedral molecules^23–25^ and planar boron clusters^1,26–28^. However, if we can develop a theory akin to the organic Kekulé model^29^ for boron materials to explain their stabilities and properties without extensive quantum calculations is still an open question. Here, based on the resonance of 3c-2e and 2c-2e bonds in triangular lattice-based flat boron materials, we present such a theory. With this theory, the bonding configurations and properties of various boron materials can be easily derived and understood. Besides the advance of the basic knowledge of boron chemistry and the understanding of the experimental puzzles, it provides an efficient tool for the design and synthesis of various boron-based materials.

## Results

### A sigma resonance theory for flat triangular boron materials

**Fig. 1:**
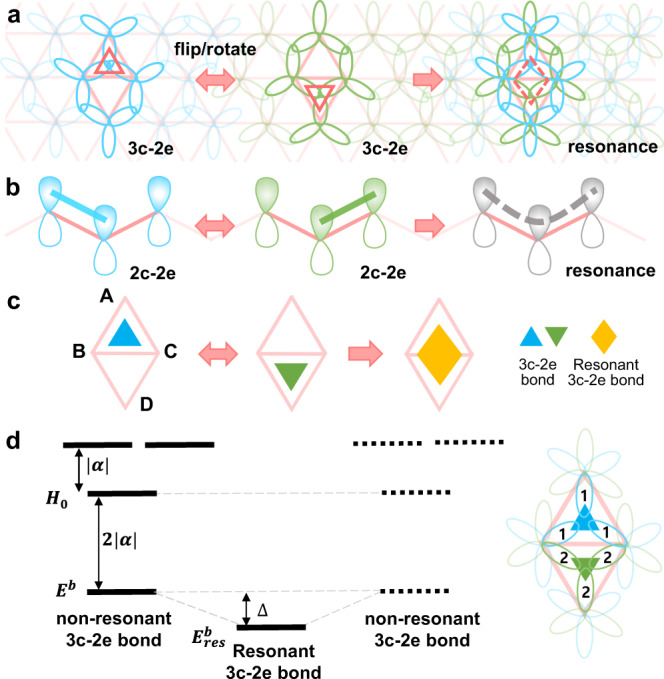
A resonance model for alternating 3c-2e bonds in a triangular boron lattice. **a** In a triangular boron network (pink), the resonance of alternating 3c-2e σ bonds in neighboring triangles leads to a diamond-shape resonant 3c-2e bond. **b** In carbon materials, the resonance of alternating 2c-2e π bonds leads to a resonant bond. **c** A simplified model of two alternating 3c-2e bonds in a diamond unit consisting of four boron atoms denoted as **A**, **B**, **C**, and **D**. **d** The energy levels of the linear combination of atomic orbitals (LCAO) of three sp^2^ orbitals of a triangular boron unit as that shown in Ref. ^19^. The lowest energy state, ${{E}^{b}=H}_{0}-2|\alpha {{{{{\rm{|}}}}}}$, corresponds a 3c-2e bond, where ${H}_{0}$ is the Coulomb integral and α is the exchange integral of two neighboring B atoms with the same sp^2^ σ orbital alignment. The resonance of the two alternating 3c-2e bonds further reduces the energy from ${E}^{b}$ to ${E}_{{res}}^{b}$ by $\triangle=\frac{4}{3}\left|\beta \right|$, where *β* is the exchange integral of two neighboring B atoms with opposite sp^2^ σ orbital alignments.

A flat triangular boron network is presented in Fig. 1a, where each sp^2^ hybridized B atom has three 3c-2e σ bonds with its six neighboring B atoms. There are two possible bonding configurations for the triangular network, one in which 3c-2e σ bonds are formed by the overlapping of three sp^2^ orbitals in the upper triangles (blue) and the other the lower triangles (green). Each 3c-2e bond in each configuration is localized and only half of the two-dimensional (2D) space (triangles) can be filled by 3c-2e bonds. Like the resonance of π bonds in benzene molecules we expect the resonance and delocalization of these 3c-2e bonds in the triangular lattice to greatly reduce the energy of the system (Fig. 1a). An analogue of the 3c-2e σ bond resonance in the triangular boron lattice is the bonding configurations of sp^2^ hybridized hydrocarbon materials, where two alternating two-center two-electron (2c-2e) π bonds resonate to form a more stable resonant bond (Fig. 1b). To note that here in this paper, we consider all the boron atoms in the flat triangular lattice-based boron materials are sp^2^ hybridized, leading to the flatness of borophene, which has been acknowledged in literatures of various 2D boron sheets, boron fullerenes, and etc^6,8,17^.

To understand the resonance between the 3c-2e bonds quantitatively, we calculate the energies of the orbitals in a sp^2^ hybridized B_4_ unit before and after resonance using a linear combination of atomic orbitals (LCAO) method^19^ (see Supplementary Note 1 and Supplementary Fig. 1). As presented in Fig. 1c, d, the energy of the 3c-2e bonding state in the upper triangle or the lower triangle is ${E}^{b}={H}_{0}-2{{{{{\rm{|}}}}}}\alpha {{{{{\rm{|}}}}}}$, where *H*_0_ is the single-electron Coulomb integral (one center Hamiltonian), and *α* is the exchange integral (two center Hamiltonian) of the neighboring B atoms with the same sp^2^ σ orbital alignment (Fig. 1d); after taking the resonance into consideration, the bonding energy is further reduced by $\triangle=\frac{4}{3}|\beta|$, where *β* is the exchange integral of the neighboring B atoms with opposite sp^2^ σ orbital alignments (see Supplementary Note 1 for details). The resonant bonding orbital is uniformly distributed at the central area of the diamond-shape unit (Fig. 1c). As shown in Supplementary Note 1, we estimate that exchange integral of two neighboring B atoms with opposite σ orbital alignments is smaller than that with the same σ orbital alignments, or $\left|\beta \right|\sim \frac{2}{3}|\alpha|$. Therefore, the resonance of two 3c-2e bonds in two neighboring triangles can significantly stabilize the system by $\frac{8}{9}{{{{{\rm{|}}}}}}\alpha {{{{{\rm{|}}}}}}$ through delocalizing the two σ bonding elections within a B_4_ unit.

### A bonding model based on the resonance theory and its verification

**Fig. 2:**
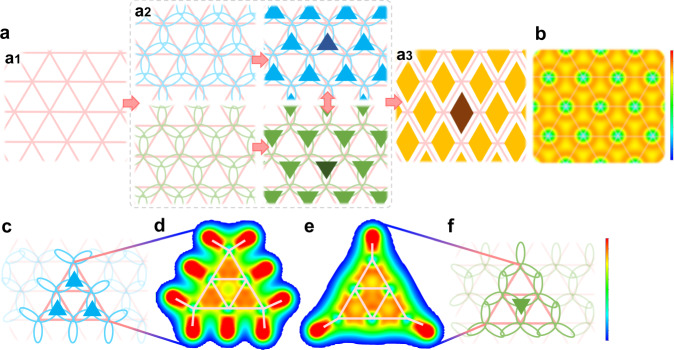
Resonant and non-resonant states of 3c-2e σ bonds in the triangular boron lattice. **a** Two σ resonance contributing configurations of a triangular boron sheet, which correspond to upwards and downwards 3c-2e bonds or triangles, respectively (a2), and one corresponding resonance hybrid structure, in which the resonance orbitals are shown by diamonds (a3). **b** The valence electron charge density (VECD) map of the perfect triangular boron sheet shows the delocalization of 3c-2e σ bonds in the sheet plane. The intensity scale bar is from 0.02 to 0.15 e/Bohr^3^ (blue to red). **c-f** σ bonding configurations of (**c**) B_6_H_9_^3-^, (**f**) B_6_H_3_ clusters based on our analysis, and (**d**, **e**) the VECD maps of them in the boron plane. The intensity scale bar is from 0.02 to 0.165 e/Bohr^3^ (blue to red).

**Fig. 3:**
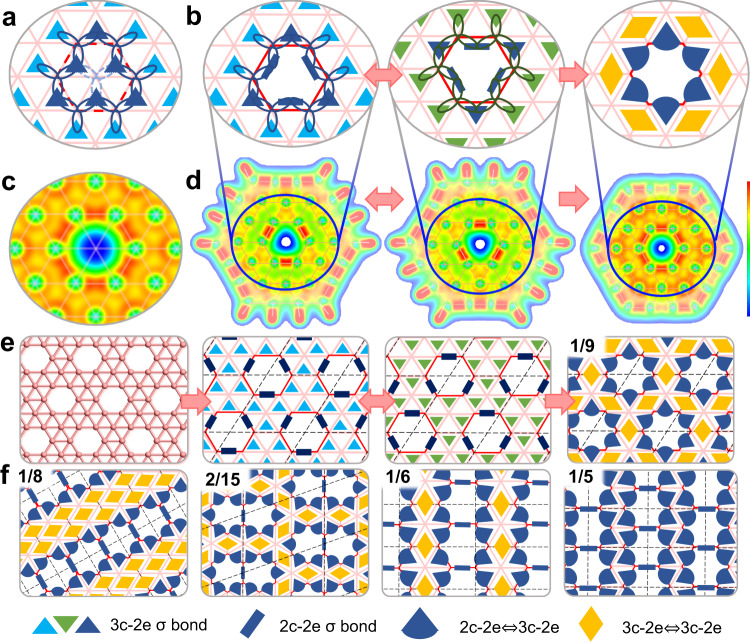
σ bonding configurations of flat boron sheets with hexagonal holes, and those of various borophene isomers. **a**–**c** Introducing a hexagonal hole into a flat triangular boron network (**a**) eliminates three 3c-2e σ bonds and (**b**) adds three 2c-2e σ bonds to the system. There are two identical bonding configurations with alternating 3c-2e and 2c-2e bonds and their resonance leads to six symmetrical bonds, as proved by (**c**) the VECD map. **d** VECD maps of B_36_H_12_ and B_36_ clusters demonstrate the existence of two identical bonding configurations containing three 3c-2e and three 2c-2e σ bonds before resonance, and that of six symmetrical bonds after resonance. **e** Atomic structure of α-borophene with its two identical σ bonding configurations before resonance and the one after resonance. **f** The resonant σ bonding configurations of borophene isomers with different hole ratios η. The intensity scale bar of (**c**) is from 0.02 to 0.15 e/Bohr^3^ (blue to red), and that of (**d**) is 0.02 to 0.16 e/Bohr^3^.

We further propose a bonding model, which, combining with above resonance theory, can intuitively describe the electronic structures of the triangular lattice-based boron sheets. The bonding model comes with three basic assumptions, i) all B atoms are sp^2^ hybridized; ii) B atoms form either 3c-2e or 2c-2e bonds; iii) all the B atoms meet the Octet rule or each B atom shares eight electrons (Figs. 2, 3). We will explicitly introduce our bonding model in the below section.

Let’s first consider the σ bonding in a flat triangular boron sheet (Fig. 2). Each atom in the triangular lattice has six neighboring triangles and can form only three 3c-2e σ bonds, either in an upper arrangement or lower arrangement, which occupy only half of the neighboring triangles (Fig. 2a). In each B_4_ unit, the resonance between the states of the upper-triangle 3c-2e σ bond and the lower-triangle 3c-2e σ bond leads to a diamond-like resonant 3c-2e bond (the so-called “4c-2e” bond in literature^4,30^). In the triangular boron network, each upper-triangle 3c-2e σ bond could resonate with either one of the three neighboring lower-triangle 3c-2e σ bonds (Fig. 2a and see Supplementary Fig. 2). This leads to a large delocalized σ bond as shown by the valence electron charge density (VECD) map calculated by density functional theory (DFT) (Fig. 2b), which might correspond to the in-plane wide band from the projected densities of states (PDOSs) in literature^8^, just as the large π bond in graphene^31,32^. It is important to note that the 4c-2e orbital is a low energy resonant state of the two alternating 3c-2e bonds according to the theory presented in this study. Although 4c-2e or nc-2e (*n* > 4) orbitals has been widely adopted in literatures based on the distribution of orbitals in boron materials, the origin of them is still unclear and they cannot be directly adopted to explain the stabilities of different boron materials. For example, directly counting the number of electrons based on the multi-centered orbitals will lead to excessive bonding electrons (> 8) on some B atoms and thus against the octet rule^5,28,30^. While in this work, using 2c-2e and 3c-2e bonds as the building blocks, we show that every B atom in the triangular lattice-based boron sheets fulfills the octet rule and the high stability of 2D boron materials is, therefore, explained. All these nc-2e (*n* > 3) bonds are resonant states of a certain number of 2c-2e and 3c-2e bonds and depends on the specific atomic structure of the 2D boron sheets. The detailed LCAO exploration of these nc-2e bonds requires more extensive research and is beyond the scope this study.

In such a sp^2^ hybridized triangular boron sheet with *N* boron atoms, all the 3*N* σ type orbitals (i.e., the sp^2^ orbitals) and *N* π type orbitals (i.e., the *p*_*z*_ orbitals) must be filled by 3*N* valence electrons to meet the Octet rule. Among them, 2*N* electrons fill *N* 3c-2e σ bonds consisting of 3*N* σ orbitals (Fig. 2a). Therefore, the σ electron-to-orbital occupation ratio is ${N}_{e}^{\sigma }/{N}_{o}^{\sigma }=2/3$, indicating that the σ system of a triangular boron lattice is electron-deficient^19^. While *N* electrons fill *N*/2 2c-2e π bonds consisting of *N* π orbitals, so the π electron-to-orbital occupation ratio is ${N}_{e}^{\pi }/{N}_{o}^{\pi }=1$. As will be shown later, the balance between occupations in σ and π systems is critical for the stability of borophene isomers. The small σ occupation ratio and the large π occupation ratio in the 2D material result in the instability of the sp^2^ hybridized single-layer triangular borophene compared to the most stable borophene isomer, the α-borophene. To note that above analysis is based on simple assumptions of molecular orbital theory^19^ and therefore the conclusions drawn from the theory are qualitative. Such as, in a 2D boron material, the orbitals are no longer discrete and thus it is very likely that some electrons could flow from the π system to the “electron-deficient” σ system, leading to the overfilling of anti-bonding σ orbitals. In previous DFT calculations^8^, the overfilling of σ orbitals has been clearly shown in the PDOS plots.

To further verify the proposed bonding model for the flat triangular boron sheet, we plot the bonding configurations of two boron clusters, B_6_H_9_^3-^ and B_6_H_3_ in Fig. 2c–f, showing the non-resonant states by proper hydrogenation. According to our model, in B_6_H_9_^3-^, the central triangle is empty while each of the three neighboring triangles accommodates a 3c-2e σ bond (Fig. 2c). For B_6_H_3_, the central triangle accommodates a 3c-2e σ bond and the three neighboring triangles are empty while the three edges of the B_6_H_3_ cluster are occupied by 6 2c-2e σ bonds (Fig. 2f). VECD maps of B_6_H_9_^3-^ and B_6_H_3_ clusters produce close analogues to those predicted by the bonding configuration model (Fig. 2d, e, and see Method) and thus, combining with the resonant state, validate the resonance model in this study.

It is well-known that vacancies or hexagonal holes can stabilize borophene^11,13^. Here we consider the bonding configurations of borophenes with hexagonal holes. As shown in Fig. 3a, b, removing one B atom from a triangular boron lattice eliminates three 3c-2e σ bonds, while the dangling orbitals of the six edge B atoms around the hole form three 2c-2e σ bonds spontaneously. The formation of the three 2c-2e bonds eliminates the six dangling orbitals, and thus stabilizes the hexagonal hole. Along with the resonance of the 3c-2e bonds in the surrounding triangular lattice, the resonance of the two identical bonding configurations consisting of three 3c-2e bonds and three 2c-2e bonds around the hexagonal hole further stabilizes the hole (Fig. 3b). VECD map at the hole vicinity (Fig. 3c) clearly shows a uniformly distributed high σ electron density at the edges of the hole, indicating the existence of the resonant state for the 3c-2e and 2c-2e bonds. The existence of three 3c-2e and three 2c-2e σ bonds before resonance, and that of six symmetrical hybrid bonds after resonance, can be clearly seen in the VECD maps of the partially hydrogenated and pristine B_36_ clusters (Fig. 3d). It is worth noting that the predicted σ bonding configuration of the pristine B_36_ cluster (see Supplementary Fig. 3) perfectly matches the result of the chemical bonding analysis presented in Ref. ^4^.

Based on above analysis and mimicking the Kekulé structure in organic chemistry, we summarize three rules for drawing the σ bonding structure of a 2D boron sheet: i) 2c-2e bonds (blue stick) appear between adjacent holes; ii) 2c-2e/3c-2e resonant bonds (blue sectors) appear at the edges of a hole; iii) as many as possible 3c-2e/3c-2e resonant bonds (yellow diamond) fill the rest of the triangular lattice. These three rules allow us to draw the σ bonding configurations of any flat boron material intuitively. Taking α-borophene as an example, the resonance of two equivalent bonding configurations leads to the resonance hybrid structure shown in Fig. 3e. Similarly, σ bonding configurations of some explored borophene isomers with various hole concentrations are shown in Fig. 3f and Supplementary Fig. 4. All these bonding configurations are validated by the VECD maps at the hole vicinity as shown in Supplementary Fig. 5.

### Stabilities and electronic structures of 2D boron materials

**Fig. 4:**
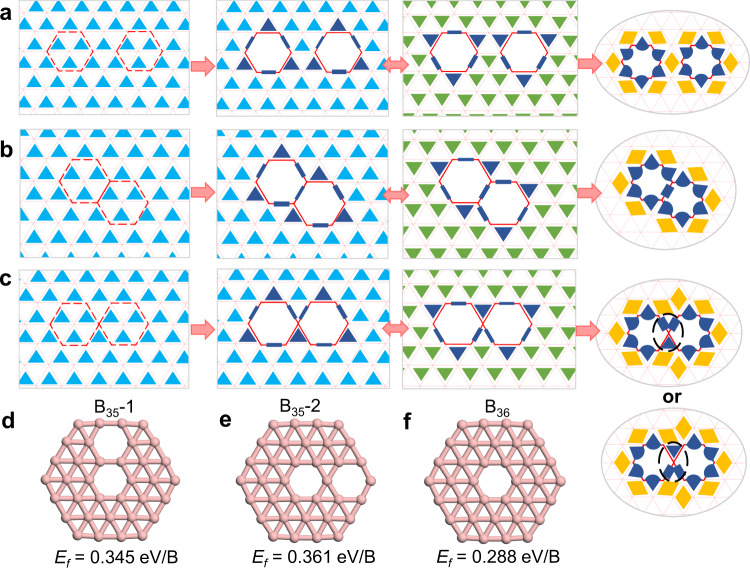
σ bonding analysis of three different double-hole structures in a 2D boron sheet. **a**-**c** σ bonding configurations of (**a**) two separated hexagonal holes, (**b**) two hexagonal holes with the shoulder-by-shoulder structure, and (**c**) two hexagonal holes with the head-to-head structure. **d**-**f** Optimized structures of (**d**) B_35_−1, (**e**) B_35_−2, and (**f**) B_36_ clusters, and their formation energies calculated by DFT method.

As that in organic chemistry, a simple model of bonding is very useful for the development of new materials. Here we use several examples to show that our model can be used as an efficient tool to predict the stability and properties of boron materials without quantum calculations. In Fig. 4a–c, we show the bonding configurations of three different double-hole structures in a 2D triangular boron lattice. Before considering bond resonance, every B atom of these three structures shares 6 σ electrons and can fulfill the octet rule by sharing 2 extra π electrons. So, it is hard to tell the relative stabilities of them. By applying the theory of resonance, we can clearly see that the separated hole structure and the shoulder-by-shoulder structure (Fig. 4a, b) can resonate. In contrast, the head-to-head double hole structure (Fig. 4c) cannot resonate because there are two 2c-2e and one 3c-2e σ bonds connected to the central B atom between the two hexagonal holes. This implies that the head-to-head double hole structure is less stable than the other two. Such a conclusion was also drawn by extensive quantum calculations^13^. In consistent with our analysis, the head-to-head double-hole configuration has never been observed in experimentally synthesized borophenes^9,10,33^, which shows a great potential of using the theory in materials design and synthesis.

Based on above bonding and resonance analysis of the double-hole structures, we predict that a B_35_ cluster with a shoulder-by-shoulder double hole structure (Fig. 4d) should be energetically more favorable than that with a head-to-head structure (Fig. 4e). DFT calculations validate our prediction and their energy difference is (0.371–0.345)*35 = 0.56 eV higher. Besides, the B_35_−1 configuration has been confirmed experimentally^34^. Furthermore, the predicted resonant bonding configurations of B_35_ and B_36_ structures (Fig. 4d, f) shown in Supplementary Fig. 6 meet the electronic structures obtained by extensive quantum calculations well (Supplementary Fig. 7)^4,34^. These examples clearly show the capacity of our theory in understanding the bonding configurations, predicting the stabilities and properties of flat boron and related materials.

### Orbital occupation balance for borophene stability evaluation

The bonding analysis resolves the σ bonding configuration for flat boron materials as each B atom is guaranteed to have six σ electrons by forming three 3c-2e and/or 2c-2e σ bonds. To fulfill the octet rule, the π electron distribution in the triangular boron sheets could also be described similarly (see Supplementary Fig. 8). For perfect triangular boron sheet, i)3*N* sp^2^ σ orbitals form *N* 3c-2e in-plane σ bonds (Fig. 2a); ii) *N p*_*z*_ orbitals form *N*/2 2c-2e out-of-plane π bonds (see Supplementary Fig. 8a); iii) each B atom shares a total of eight electrons; and iv) both σ and π electrons are delocalized (see Supplementary Fig. 8b)^26,27^. Adding a hole to a triangular boron sheet transforms some 2c-2e π bonds into 3c-2e π bonds (see Supplementary Fig. 8c–e), which is the opposite of the σ system where 3c-2e σ bonds are transformed into 2c-2e σ bonds.

As described above, the triangular borophene has the σ electron-to-orbital occupation ratio of ${N}_{e}^{\sigma }/{N}_{o}^{\sigma }=2/3$ and the π electron-to-orbital occupation ratio of ${N}_{e}^{\pi }/{N}_{o}^{\pi }=1$. The formation of *Nη* hexagonal holes (where η is the hole to original B count ratio) via B atom removal removes 3*Nη* σ type orbitals and *Nη* π type orbitals. Thus, ${N}_{o}^{\sigma }=3N-3N\eta$, and ${N}_{o}^{\pi }=N-N\eta$ . The removal of *Nη* B atoms also removes 3*Nη* valence electrons. However, as 3*Nη* 3c-2e σ bonds are replaced by 3*Nη* 2c-2e σ bonds, the number of σ electrons remains as ${N}_{e}^{\sigma }=2N$ and all valance electrons removed are π electrons so ${N}_{e}^{\pi }=N-3N\eta$ . Thus, in a boron sheet with a hole ratio of *η* the σ electron-to-orbital occupation ratio increases to

1 $${N}_{e}^{\sigma }/{N}_{o}^{\sigma }=2N/(3N-3N\eta )=2/(3-3\eta )$$

while the π electron-to-orbital occupation ratio decreases to

2 $${N}_{e}^{\pi }/{N}_{o}^{\pi }=(N-3N\eta )/(N-N\eta )=(1-3\eta )/(1-\eta )$$

This implies that the introduction of holes can alleviate the electron-deficiency of the σ system by π-to-σ self-doping^8,13,15^ and therefore stabilizes the triangular lattice. It is worth noting that our analysis of the hole bonding configuration and electron counting well explains why the number of σ electrons remains the same in 2D boron structures with different hole ratios^15^.

Extensive ab initio calculations have found that the stability of a neutral boron sheet depends on the hole concentration η with those having η ~ 1/9 being the most stable^11^. Here we introduce an occupation imbalance factor (δ) of a neutral boron material as

3 $${{{{{\rm{\delta }}}}}}={{{{{\rm{|}}}}}}{N}_{e}^{\pi }/{N}_{o}^{\pi }-{N}_{e}^{\sigma }/{N}_{o}^{\sigma }{{{{{\rm{|}}}}}}={{{{{\rm{|}}}}}}\left(1-9\eta \right)/\left(3-3\eta \right){{{{{\rm{|}}}}}}$$

**Fig. 5:**
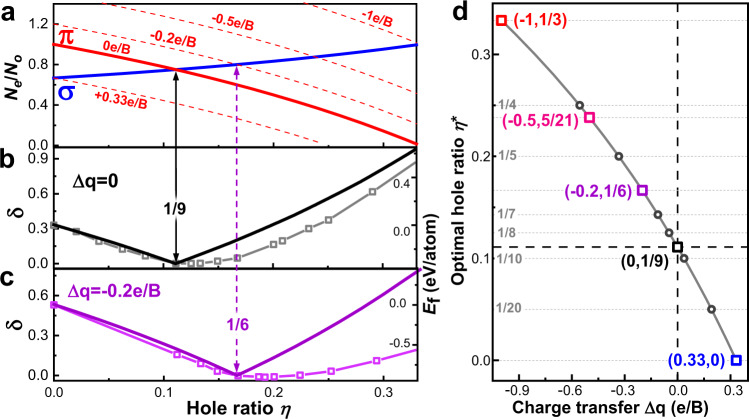
Borophene stability analysis at different doping levels. **a** Electron-to-orbital occupation ratios ${N}_{e}/{N}_{o}$ of π (red) and σ (blue) bonding configurations of neutral (solid curves) and doped (dash curves) borophene isomers as functions of hole ratio η. **b**-**c** Occupation imbalance factor δ = |${N}_{e}^{\pi }/{N}_{o}^{\pi }-{N}_{e}^{\sigma }/{N}_{o}^{\sigma }$| of (**b**) neutral borophene (Δq=0) and (**c**) borophene with charge transfer of −0.2 e per B atom as functions of η. Borophene formation energies adapted from Ref. ^11,36^. are presented with right-*y* axes in grey and purple squares in (**b**) and (**c**), respectively. **d** Optimal hole ratio η* of borophene isomers as a function of doping level Δq. Source data are provided as a Source Data file.

Electron-to-orbital occupation ratios (${N}_{e}/{N}_{o}$) of both π (red) and σ (blue) bonding configurations, along with δ values (black) for neutral borophene isomers as functions of hole ratio η are presented in Figs. 5a and 4b, respectively. The δ reaches its minimum 0 at η = 1/9, which means that borophene with a ~1/9 hole concentration has a balanced occupation for σ and π orbitals (i.e., ${N}_{e}^{\sigma }/{N}_{o}^{\sigma }={N}_{e}^{\pi }/{N}_{o}^{\pi }$) (Fig. 5b). A strong correlation between the δ and the formation energies of various borophene isomers (grey squares in Fig. 5b, from Ref. ^11^) is clearly seen. Thus, we conclude that the occupation imbalance factor can be regarded as an indicator of the stability of flat boron materials, and any boron sheet with a small δ value (e.g., δ < 0.1) might be considered a stable phase.

We would like to note that δ is the orbital occupation (${N}_{e}/{N}_{o}$) difference between in-plane (σ) and out-of-plane (π) orbitals, which evaluates the difference of the electron deficiency in the two electron systems. δ = 0 indicates that the orbital occupation rates of σ and π are equal. The analysis can also be applied to other 2D materials. For example, all the bonding orbitals (both σ and π) of neutral graphene are fully occupied and all the anti-bonding orbitals are unoccupied, and thus δ = 0 for the neutral graphene. This implies that δ can be considered as an indicator of electron occupation in bonding and antibonding orbitals or, equivalently, the stability of the materials. It is interesting that the analysis in Ref. ^15^, which is based on quantum calculations, also shows 2D borophene structures with a hole ratio of 1/9 is more stable than others and it has a 1/3 π-to-σ electron ratio. We need to mention that above analysis is based on the assumption that the number of σ electrons in boron sheets with different hole ratios is a constant, which is reasonable for borophene with optimal hole ratio, η ~ 1/9. While, for borophene with small (η ~ 0) or very large hole ratios (i.e., η ~ 1/3), large deviation between the theory and the DFT results may exist.

The occupation balance discussed above can also be used to explain the formation of borophene isomers with various hole concentrations on metal substrates^12,33,35^. Previous studies showed that the PDOSs of stable borophene isomers^8,13,15,18^, such as α-borophene (see Supplementary Fig. 9), always have significant gaps for σ orbitals near the Fermi level and those for π orbitals are conducting. We therefore reasonably assume that the doped charge goes to π orbitals, and thus Eq. (2) and Eq. (3) become

4 $${N}_{e}^{\pi }/{N}_{o}^{\pi }=(1-3\eta )/\left(1-\eta \right)+\triangle q$$

and

5 $${{{{{\rm{\delta }}}}}}={{{{{\rm{|}}}}}}{N}_{e}^{\pi }/{N}_{o}^{\pi }-{N}_{e}^{\sigma }/{N}_{o}^{\sigma }{{{{{\rm{|}}}}}}={{{{{\rm{|}}}}}}\left(1-9\eta \right)/\left(3-3\eta \right)+\triangle q{{{{{\rm{|}}}}}}$$

where Δq is the charge transfer per B atom from the substrate to the borophene isomer. Therefore, δ can be tuned by the doping level Δq. As shown in Fig. 5a, c, a doping of −0.2e per B atom leads to a shift in occupation ratio of π orbitals and thus a shift in the optimal hole ratio η* (where δ = 0) to 1/6. As expected, the calculated formation energies^36^ clearly indicate that electron doping tends to favor borophene isomers with larger hole concentration (purple squares in Fig. 5c). The optimal hole ratio η* as a function of Δq is shown in Fig. 5d, from which we can predict the optimal hole ratios for doped borophene isomers, and the result matches the analysis in Ref. ^15^. though different approaches have been used. The configuration-based analysis agrees well with the experimental results and those obtained through extensive quantum calculations^12,18,33,35–37^. For example, on a Ag substrate, slight charge transfer stabilizes borophene isomers with η = 1/6 to 1/5^33,35^; while on a Al surface, significant electron doping leads to the high stability of hexagonal borophene with η = 1/3^12^. According to our prediction shown in Fig. 5d, synthesis of flat triangular boron sheet is possible if the substrate can take away ~ 0.33 electrons for each B atom.

## Discussion

We need to point out that although the analyses based on our theory of bonding configuration are qualitative, it provides clear physical or chemical pictures to understand the results of quantum calculations. By combining the bonding configurations provided by our theory with the quantum computations, we can understand the stability and properties of boron materials in quantity and with high efficiency. For example, although the proposed occupation imbalance factor δ is capable of roughly estimating the relative stabilities of different borophene isomers based on their doping levels on a substrate, extensive DFT calculation are required to fully address the formation of various borophenes on different substrates^9,36^.

We highlight that the assumptions for the whole theory of resonance and the bonding configurations are simply the sp^2^ hybridization, the formation of either 3c-2e or 2c-2e bonds, and the Octet rule. In other words, our model is also applicable to many other boron materials. For example, bonding configurations of boron fullerene cages and quasi-flat boron clusters can be easily drawn with these proposed rules (see Supplementary Figs. 6, 10). It is clear that the predicted bonding configuration agrees with the quantum calculation results (Supplementary Fig. 10c)^4,34^. Subsequent electron and bond analysis of the B_80_ cage shows that it has balanced occupation ratios of σ and π orbitals (both are 3/4), which accounts for its superior stability, and also demonstrates the effectiveness of our theory. More importantly, it also provides an opportunity to study the bonding features of other boron bulky structures like the bulk α boron as shown in Supplementary Fig. 10d, h.

In summary, we have developed a theory of bonding in triangular lattice-based boron materials via σ bond resonance, mimicking π bond resonance and the Kekulé model in organic materials. This theory allows us to explicitly describe the electronic structures of boron materials without extensive quantum calculations. With the assumption of orbital occupation balance we have successfully explained the high stability of borophene with 1/9 hole concentration and the charge doping dependent optimum hole concentration for borophene isomers.

## Methods

### Density functional theory (DFT) calculations for the valence electron charge density (VECD)

Valence electron distribution denotes the nature of the chemical bonding among atoms by summing the valence electron charge density, having removed the contribution from neutral spherical atoms. In this paper, we map only the valence electron charge density in the plane (z = 0) to show the σ bonding features by DFT calculations performed with Vienna ab initio Simulation Package (VASP)^38,39^ using projected augmented wave (PAW) method^40^ and generalized gradient approximation (GGA)^41^ energy for the exchange-correlation interactions. All the boron flat clusters or the borophene isomers were first optimized and then the valence electron charge density maps of these optimized structures in the plane were calculated. DFT-D3 method^42^ was employed to describe the van der Waals interaction. The plane-wave cutoff energy was set as 400 eV. The Brillouin zone was sampled using Monkhorst-Pack k-mesh^43^ with a separation criterion of 0.02. Criteria for energy and force convergence were 10^−4^ eV and 10^−2^ eV/Å, respectively.

### Reporting summary

Further information on research design is available in the Nature Portfolio Reporting Summary linked to this article.

## Supplementary information

Supplementary Information

Reporting Summary

## Data Availability

All the data supporting the findings of this study are provided within the article and its Supplementary Information. Source data are provided as a Source Data file. Source data are provided with this paper.

## Source data

Source Data
