# Supplementary material for: Theory of sigma bond resonance in flat boron materials
Source: Nat Commun. 2023 Mar 31;14:1804. doi: 10.1038/s41467-023-37442-8 (PMC10066189; doi:10.1038/s41467-023-37442-8)
Supplement: Supplementary file 1 — Supplementary Information [file 41467_2023_37442_MOESM1_ESM.pdf]

Supplementary Information

Theory of sigma bond resonance in flat boron materials

Lu Qiu et al

Supplementary Note 1

Linear combination of atomic orbitals of B_4 unit with resonance

In principle, we need to deal with 12 orbitals (thus a 12×12 matrix) that can form σ bonds, as every B atom (Supplementary Fig. 1a) has 3 sp^2 hybrid atomic orbitals (AOs) in the B_4 unit of D_{2h} point group (Supplementary Fig. 1b and 1c). A close inspection shows that only 4 AOs can form 3c-2e bonds (Supplementary Fig. 1d and 1e), while the other 8 AOs form either 2c-2e bonds or the dangling bonds (non-bonding). Therefore, we just focus on the 4 AOs, labeled as $|\phi_A\rangle$, $|\phi_B\rangle$, $|\phi_C\rangle$, and $|\phi_D\rangle$, that can form 3c-2e bond, which is of specific interest in this study.

The *i*th molecular orbital (MO) $|\psi_i\rangle$ can be constructed via linear combination of atomic orbitals (LCAO), i.e.,

$$|\psi_i\rangle = \sum_j c_{ij}|\phi_j\rangle, \tag{S1}$$

in which j loops over A, B, C and D, and c_{ij} is the coefficient. Adapting Eq. (S1) in Schrödinger Equation, $\hat{H}|\psi_i\rangle = E_i|\psi_i\rangle$, in which $\hat{H}$ is the Hamiltonian operator and E_i is the eigenvalue of $|\psi_i\rangle$. Applying linear variation leads to the pseudo-eigenvalue equation[1]

$$HC = ESC, \tag{S2}$$

in which H is the Hamiltonian matrix with element $H_{ij} = \langle\phi_i|\hat{H}|\phi_j\rangle$, E is the eigenvalue, C is the coefficient, and S is the overlap matrix with element $S_{ij} = \langle\phi_i|\phi_j\rangle$. We next apply Hückel approximation, i.e.,

$$H_{ij} = \begin{cases} H_0, & i = j \\ \alpha, & overlap\ between\ |\phi_i\rangle\ and\ |\phi_j\rangle \\ 0, & otherwise \end{cases} \quad \text{and} \quad S_{ij} = I = \begin{cases} 1, & i = j \\ 0, & i \neq j \end{cases} \tag{S3}$$

Eq. (S2) becomes eigenvalue equation, i.e.,

$$HC = EC \tag{S4}$$

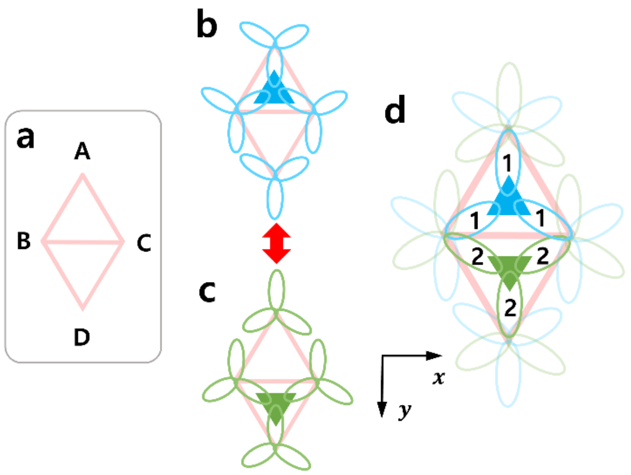

Supplementary Figure 1: (**a**) B_4 unit with four B atoms. (**b-c**) Two resonant contributing bonding configurations of the B_4 unit with the three sp^2 hybrid atomic orbitals (AOs) on each B atom labeled and flipped from (b) to (c). (**d**) The resonant orbital configuration which can form either up-triangle 3c-2e or down-triangle 3c-2e bonds.

For the non-resonance treatment, the two bonding configurations in Supplementary Fig. 1b and 1c are equivalent. Taking the bonding configuration in Supplementary Fig. 1b as an example, we observe that $|\phi_A\rangle$, $|\phi_B\rangle$, and $|\phi_C\rangle$ overlap on the upper triangle, while $|\phi_D\rangle$ is dangling. Using Hückel approximation in Eq. (S3), the corresponding non-resonance Hamiltonian matrix $H_{non-res}$ of Supplementary Fig. 1b can be written as

$$H_{non-res} = \begin{pmatrix} H_0 & \alpha & \alpha & 0 \\ \alpha & H_0 & \alpha & 0 \\ \alpha & \alpha & H_0 & 0 \\ 0 & 0 & 0 & H_0 \end{pmatrix}. \tag{S5}$$

Note that Eq. (S5) consists of two sub-matrices of 3×3 and 1×1, and the latter gives the non-bonding orbital $|\psi^{nb}\rangle = |\phi_D\rangle$ with orbital energy $E^{nb} = H_0$ directly. Applying Eq. (S4) on the remaining 3×3 sub-matrix, we have the energy $E^b = H_0 + 2\alpha$ for the

3c-2e bonding orbital and two degenerate anti-bonding orbitals with energy $E^{ab} = H_0 - \alpha$. The bonding orbital for the 3c-2e bond takes the form $|\psi^b\rangle = \frac{1}{\sqrt{3}}(|\phi_A\rangle + |\phi_B\rangle + |\phi_C\rangle)$, the same as was defined in the pioneer work of Dilthey on the multicenter orbital[2].

There are defects in the above non-resonance treatment as it does not consider that the two contributing configurations are indistinguishable, and the symmetry of the constructed MOs does not match that of the B_4 unit. Therefore, below we consider the resonance of the two indistinguishable state and calculate the corresponding resonance energy.

As shown in the first part (Supplementary Fig. 1b), the bonding orbital of the up-triangle 3c-2e (blue) is

$$|\psi_{up}\rangle = \frac{1}{\sqrt{3}}(|\phi_{A1}\rangle + |\phi_{B1}\rangle + |\phi_{C1}\rangle), \tag{S6}$$

with the energy $E_{non-res} = H_0 - 2|\alpha|$. Similarly, we have the bonding orbital of the down-triangle 3c-2e (green) as

$$|\psi_{down}\rangle = \frac{1}{\sqrt{3}}(|\phi_{D2}\rangle + |\phi_{B2}\rangle + |\phi_{C2}\rangle), \tag{S7}$$

with the same bonding energy. To note that here the sp^2 σ orbitals forming the up-triangle 3c-2e bond (ϕ_{A1}, ϕ_{B1}, and ϕ_{C1}) and those (ϕ_{D2}, ϕ_{B2}, and ϕ_{C2}) forming the down-triangle 3c-2e bond are oppositely aligned, and we use subscripted 1 and 2 to distinguish them.

To calculate the resonance energy and state between the two indistinguishable 3c-2e bonding states, we construct the Hamiltonian as

$$H = \begin{pmatrix} \langle\psi_{up}|H|\psi_{up}\rangle & \langle\psi_{up}|H|\psi_{down}\rangle \\ \langle\psi_{down}|H|\psi_{up}\rangle & \langle\psi_{down}|H|\psi_{down}\rangle \end{pmatrix}. \tag{S8}$$

Taking above 3c-2e bonding result into the Hamiltonian, we have

$$\langle\psi_{up}|H|\psi_{up}\rangle = \langle\psi_{down}|H|\psi_{down}\rangle = H_0 - 2|\alpha|, \tag{S9}$$

and

$$\langle\psi_{up}|H|\psi_{down}\rangle = \langle\psi_{down}|H|\psi_{up}\rangle = -\frac{4}{3}|\beta|, \tag{S10}$$

where $\langle\phi_{A1}|H|\phi_{B1}\rangle = \langle\phi_{A1}|H|\phi_{C1}\rangle = \langle\phi_{D2}|H|\phi_{B2}\rangle = \langle\phi_{D2}|H|\phi_{C2}\rangle = \langle\phi_{B2}|H|\phi_{C2}\rangle = -|\alpha|$ is the exchange integral between two neighboring B atoms with the same σ bond orientation, $\langle\phi_{A1}|H|\phi_{B2}\rangle = \langle\phi_{A1}|H|\phi_{C2}\rangle = \langle\phi_{D2}|H|\phi_{B1}\rangle = \langle\phi_{D2}|H|\phi_{C1}\rangle = -|\beta|$ is the exchange integral between two neighboring B atoms with opposite σ bond orientations, and other exchange integrals are set as 0 due to the minor overlapping between these orbitals. Solving the secular equation, we have the resonant bonding state with the energy of $E_{res} = H_0 - 2|\alpha| - \frac{4}{3}|\beta|$, and thus we have the resonance energy $-\frac{4}{3}|\beta|$.

Here we estimate $|\beta|$ by the orthogonality of the rotated sp^2 orbitals. Take the bonding between ϕ_{D2} and ϕ_{B1} as an example. The two orbitals can be written as a linear combination of $|2s\rangle$, $|2p_x\rangle$ and $|2p_y\rangle$ orbitals:

$$|\phi_{B1}\rangle = \frac{1}{\sqrt{3}}|2s\rangle + \sqrt{\frac{2}{3}}(\frac{\sqrt{3}}{2}\left|2p_x\right\rangle - \frac{1}{2}\left|2p_y\right\rangle), \tag{S11}$$

$$|\phi_{B2}\rangle = \frac{1}{\sqrt{3}}|2s\rangle + \sqrt{\frac{2}{3}}(\frac{\sqrt{3}}{2}\left|2p_x\right\rangle + \frac{1}{2}\left|2p_y\right\rangle). \tag{S12}$$

So, we have $\langle\phi_{B1}|\phi_{B2}\rangle = 2/3$. Thus, because other orbital components of B atom have minor overlapping with orbitals of D atom, we can use $\langle\phi_{D2}|H|\phi_{B1}\rangle = -|\beta| = -\frac{2}{3}|\alpha|$ as the exchange integral between B atom and D atom with different σ bond orientations. Therefore, the resonance energy of the two indistinguishable 3c-2e bonds is estimated to be $\frac{8}{9}|\alpha\ |$, which is significant.

Supplementary Figures

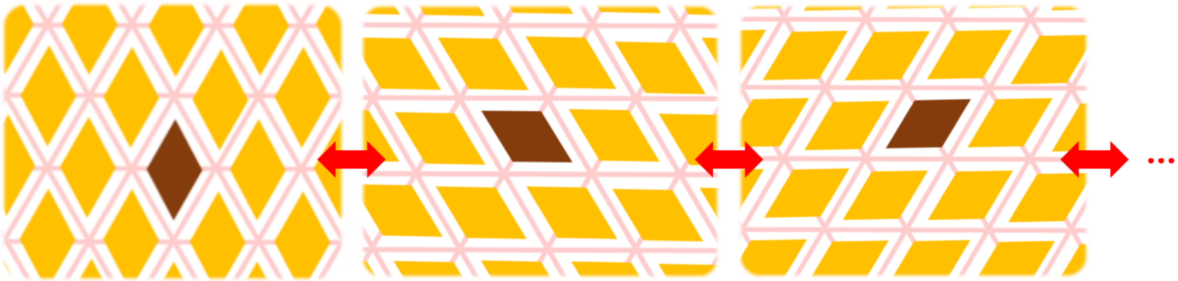

Supplementary Figure 2: Three major resonance hybrid structures for the flat triangular boron sheet.

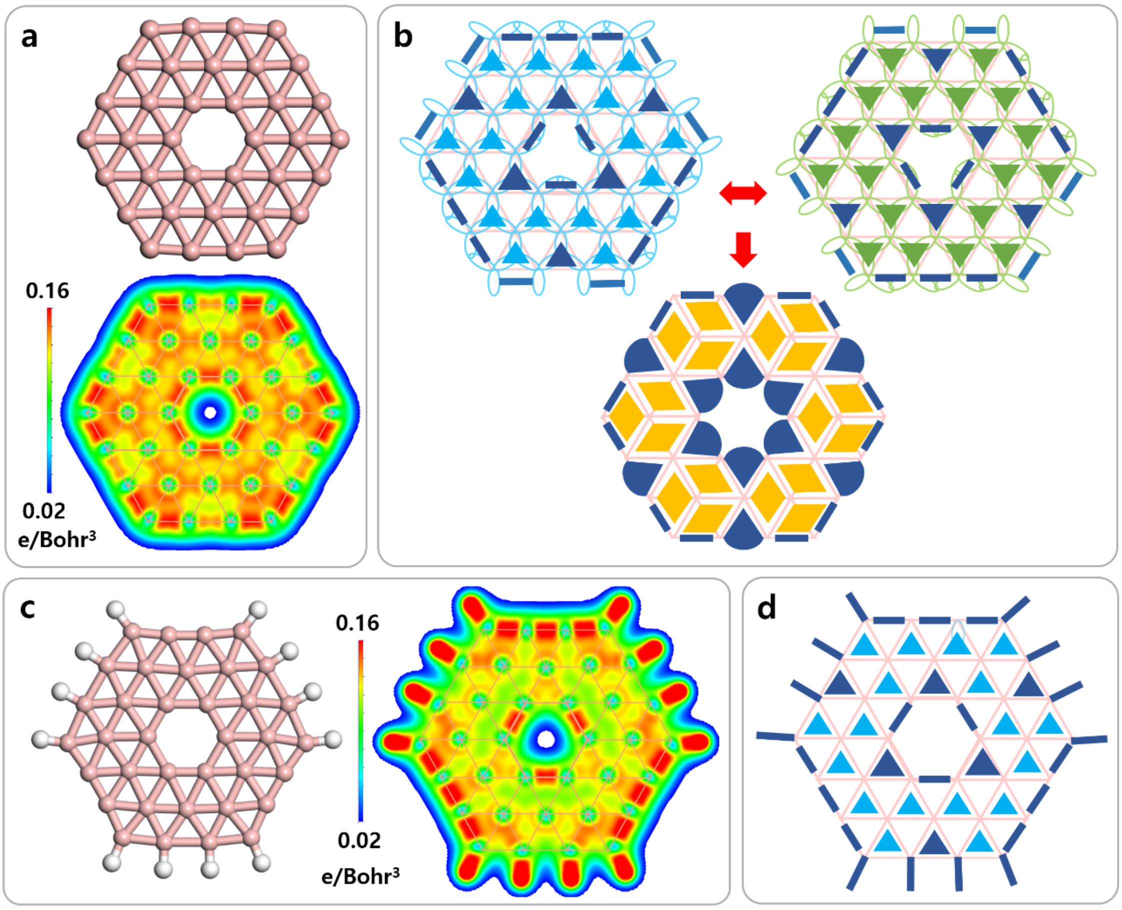

Supplementary Figure 3: Electronic structures of B_{36} and its hydrogenated clusters. Valence electron charge density (VECD) maps of (**a**) B_{36}, and (**c**) $B_{36}H_{12}$ in the cluster surface, and the corresponding (**b** and **d**) σ bonding configurations by our theory.

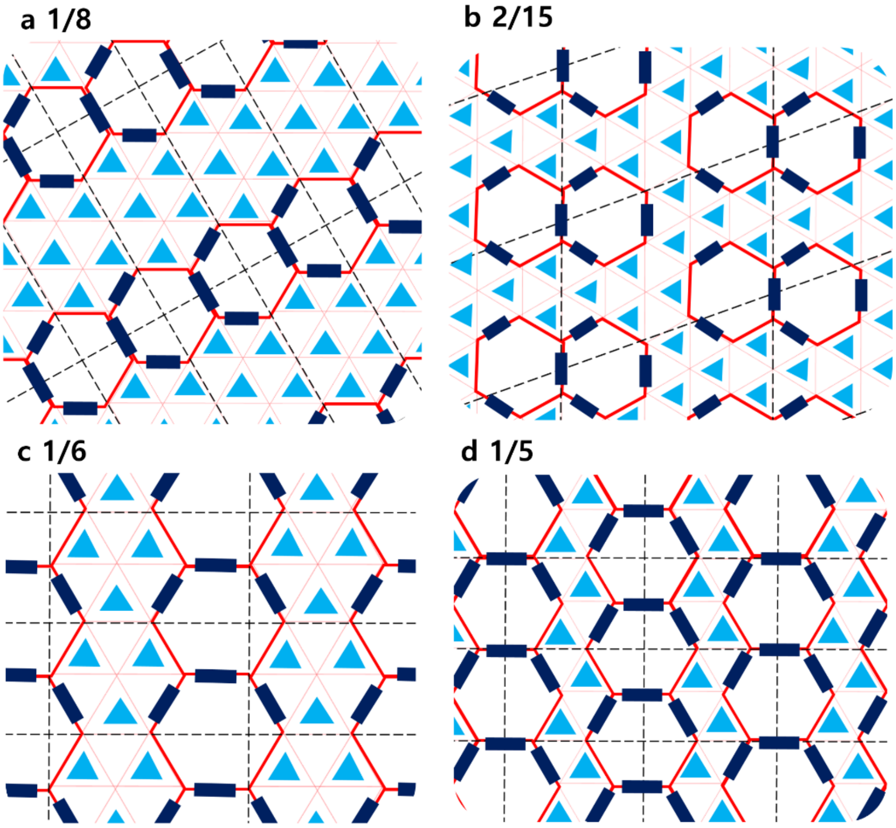

Supplementary Figure 4: σ bonding configurations of various 2D boron sheets. σ bonding configurations of 2D boron sheets with hole ratios of (**a**) 1/8, (**b**) 2/15, (**c**) 1/6, and (**d**) 1/5.

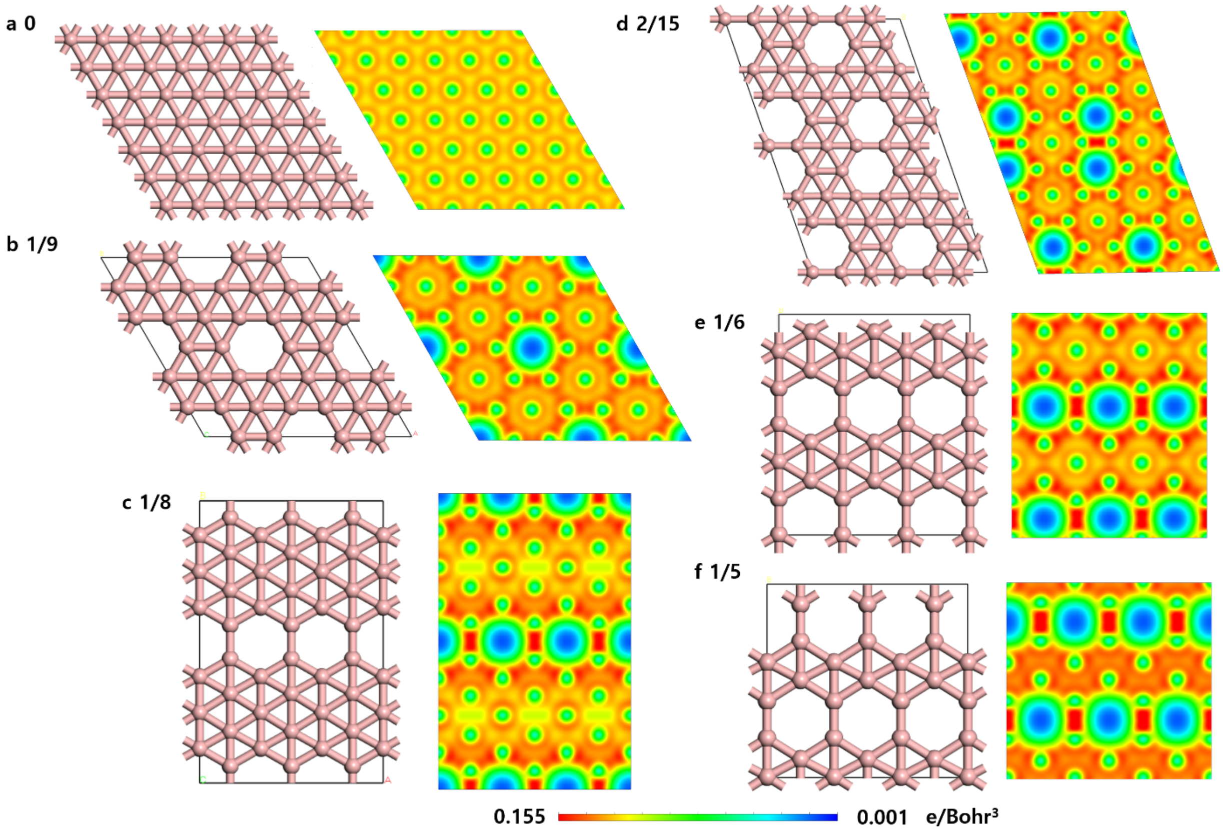

Supplementary Figure 5: Electronic structures of 2D boron sheets. VECD maps of 2D boron sheets with hole ratios of (**a**) 0, (**b**) 1/9, (**c**) 1/8, (**d**) 2/15, (**e**) 1/6, and (**f**) 1/5.

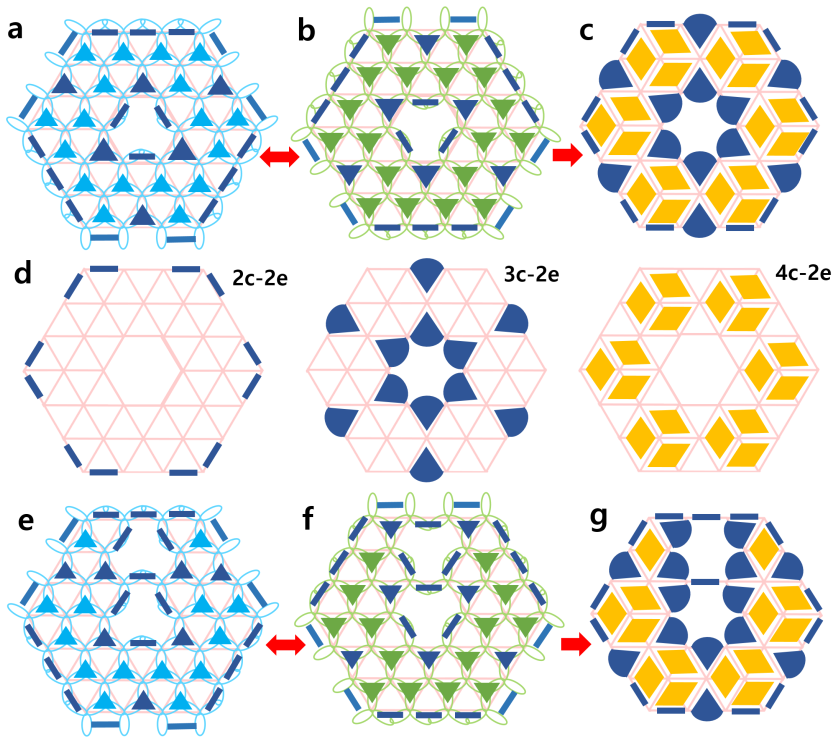

Supplementary Figure 6: σ bonding configurations of B_{36} and B_{35} clusters. σ bonding configurations of the (**a-c**) B_{36}, and (**e-g**) B_{35} clusters before and after resonance based on the proposed theory of resonance, where the resonant bonding configuration of B_{36} consists of (**d**) 2c-2e, 3c-2e, and 4c-2e bonds, respectively. The drawn resonant bonding configurations (c) and (g) match well with the AdNDP chemical bonding analysis from Ref. 5 and 6, respectively, as shown in Supplementary Fig. 7.

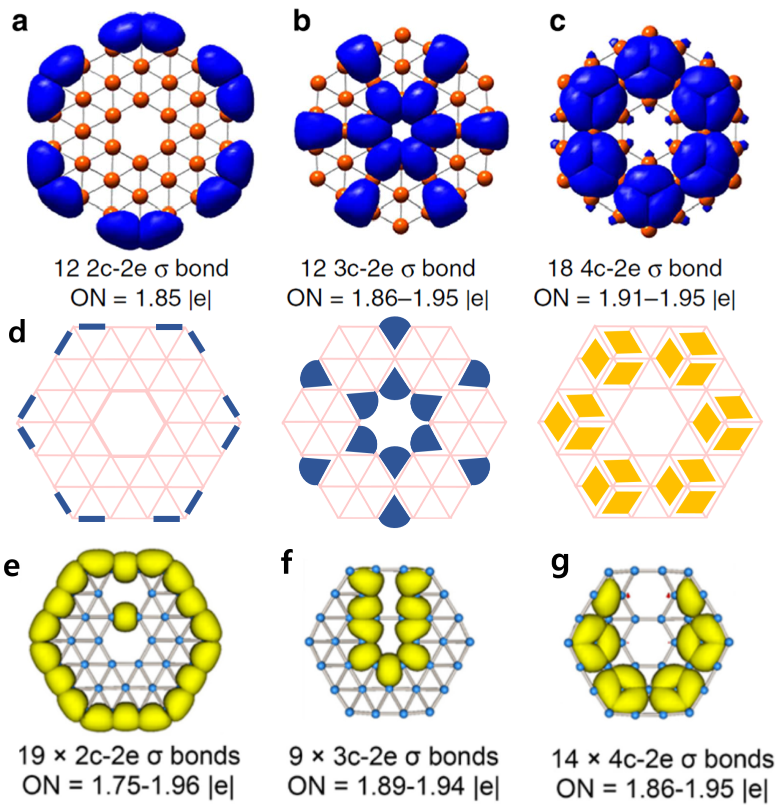

Supplementary Figure 7: Comparison of the electronic structures of B_{36} and B_{35} clusters between literature and our theory. The AdNDP chemical bonding analysis of (**a-c**) B_{36}, and (**e-g**) B_{35} clusters match well with the electronic structures predicted by our theory as shown in (**d**) and Supplementary Fig. 6. (a-c) Preprinted with permission from Ref. 5. Copyright 2014 Nature Publishing Group. (e-g) Preprinted with permission from Ref. 6. Copyright 2014 American Chemical Society.

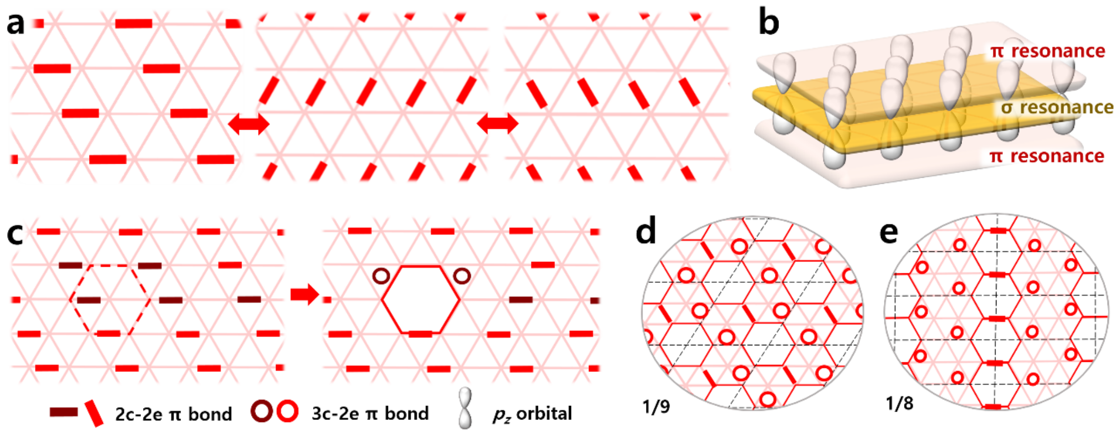

Supplementary Figure 8: π electron distribution in flat boron sheets according to the octet rule and the resonance model. (**a**) Three major resonance contributing forms of the π system in the flat triangular boron. (**b**) Schematic diagram showing both the large delocalized σ and π bonds in the flat triangular boron sheet. (**c**) Introducing a single-atom hole into the flat triangular boron network will delete 3.5 2c-2e and add 2 3c-2e π bonds to the system. (**d-e**) Representative π bonding configurations of borophene isomers with 1/9 and 1/8 hole ratios.

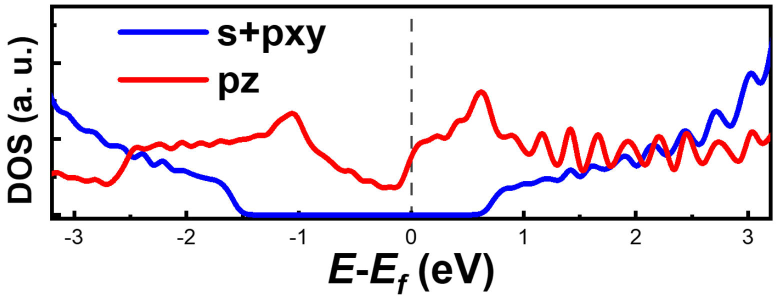

Supplementary Figure 9: Projected density of states (PDOS) for α-borophene, from which we can see that σ orbitals have a gap of ~ 2 eV near the Fermi level. Source data are provided as a Source Data file.

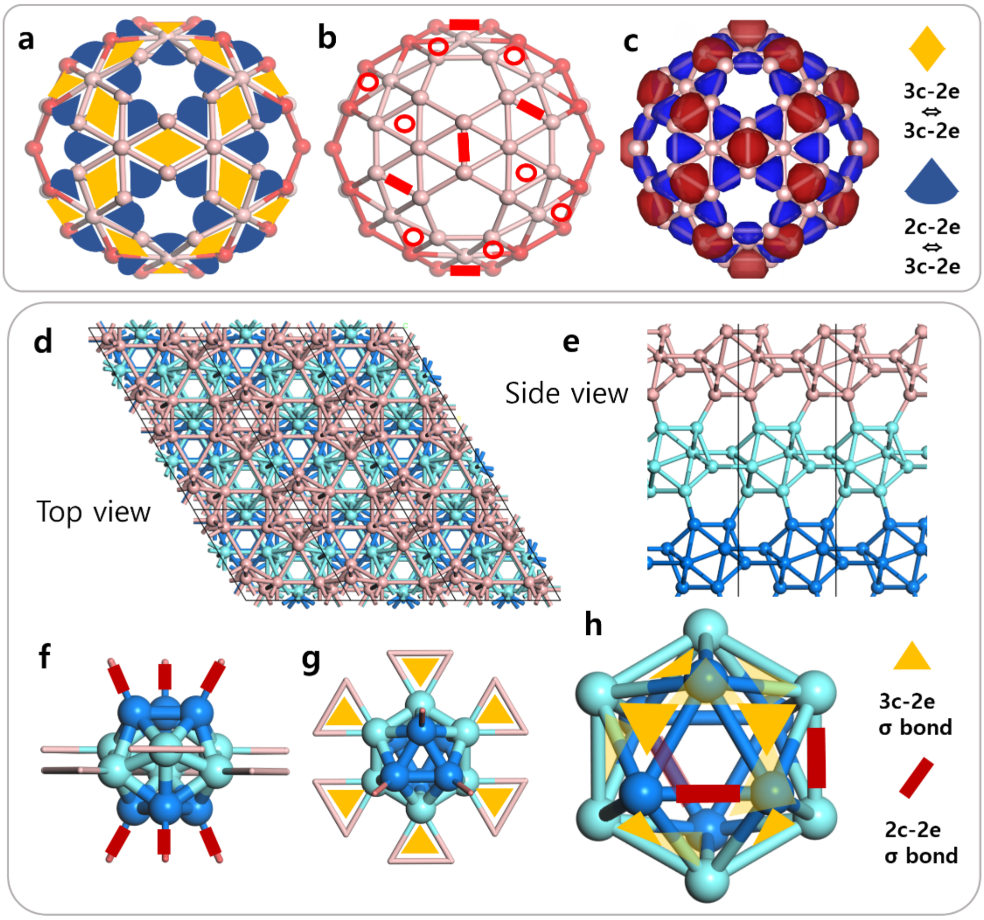

Supplementary Figure 10: Applications of the theory on other boron materials. (**a**) σ bonding configuration of a B_{80} fullerene cage. (**b**) π bonding configuration of a B_{80} fullerene cage. There are totally 80×3=240 electrons in a B_{80} fullerene cage with 60 3c-2e σ bonds, 30 2c-2e σ bonds, 20 3c-2e π bonds, and 10 2c-2e π bonds. So the electron-to-orbital ratios of σ and π systems are both 3/4. (**c**) Chemical bonding analysis of a B_{80} fullerene cage using the AdNDP method[7]. Atomic structure of bulk alpha boron from (**d**) top and (**e**) side views. (**f-h**) Focusing on the bonds of a B_{12} cluster in the bulk alpha boron, there are 6 2c-2e (f, side view) and 6 3c-2e (g, top view) inter-cluster σ bonds, and 10 3c-2e and 3 2c-2e (h, top view) intra-cluster σ bonds.

Supplementary References

1. W. Moffitt, Molecular orbitals arid the Hartree field. *Proceedings of the Royal Society of London. Series A. Mathematical and Physical Sciences* **196**, 510-523 (1949).

2. W. Dilthey, The synaptic order, a key concept to understand multicenter. *Zeitschrift für Angewandte Chemie* **34**, 596-599 (1921).

3. W. H. Eberhardt et al., The Valence Structure of the Boron Hydrides. The *Journal of Chemical Physics* **22**, 989 (1954).

4. M. Nakhaee *et al.,* Tight-binding Model for Borophene and Borophane. *Physical Review B* **97**, 125424 (2018).

5. Z. A. Piazza *et al.*, Planar hexagonal B36 as a potential basis for extended single-atom layer boron sheets. *Nature Communications* **5**, 3113 (2014).

6. W.-L. Li *et al.,* The B35 Cluster with a Double-Hexagonal Vacancy: A New and More Flexible Structural Motif for Borophene. *Journal of the American Chemical Society* **136**, 12257-12260 (2014).

7. D. Y. Zubarev, & A. I. Boldyrev, Developing paradigms of chemical bonding: adaptive natural density partitioning. *Physical Chemistry Chemical Physics* **10**, 5207–5217 (2008).
